# Electro-pharmacological profiles of two brain mitoplast anion channels: Inferences from single channel recording

**DOI:** 10.17179/excli2016-808

**Published:** 2017-04-18

**Authors:** Javad Fahanik-Babaei, Farzad Shayanfar, Naser Khodaee, Reza Saghiri, Afsaneh Eliassi

**Affiliations:** 1 Neurophysiology Research Center, Shahid Beheshti University of Medical Sciences, Tehran, Iran; 2 Neuroscience Research Center, Shahid Beheshti University of Medical Sciences, Tehran, Iran; 3 Department of Physiology, Medical School, Shahid Beheshti University of Medical Sciences, Tehran, Iran; 4 Faculty of Paramedical Sciences, AJA University of Medical Sciences, Tehran, Iran; 5 Department of Biochemistry, Pasteur Institute of Iran, Tehran, Iran

**Keywords:** mitochondria, chloride channels, single channel, intracellular ion channels, brain, mitoplast

## Abstract

We have characterized the conduction and blocking properties of two different chloride channels from brain mitochondrial inner membranes after incorporation into planar lipid bilayers. Our experiments revealed the existence of channels with a mean conductance of 158 ± 7 and 301 ± 8 pS in asymmetrical 200 mM cis/50 mM trans KCl solutions. We determined that the channels were ten times more permeable for Cl^−^ than for K^+^, calculated from the reversal potential using the Goldman-Hodgkin-Katz equation. The channels were bell-shaped voltage dependent, with maximum open probability 0.9 at ± 20 mV. Two mitochondrial chloride channels were blocked after the addition of 10 µM DIDS. In addition, 158 pS chloride channel was blocked by 300 nM NPPB, acidic pH and 2.5 mM ATP, whereas the 301 pS chloride channel was blocked by 600 µM NPPB but not by acidic pH or ATP. Gating and conducting behaviors of these channels were unaffected by Ca^2+^. These results demonstrate that the 158 pS anion channel present in brain mitochondrial inner membrane, is probably identical to IMAC and 301 pS Cl channel displays different properties than those classically described for mitochondrial anion channels.

## Introduction

Mitochondria are involved in various processes essential for cell survival, including energy production, redox control, calcium homeostasis, and physiological cell death mechanisms. Mitochondrial ion channels play a role in these processes by influencing organellar membrane potential, ROS production, volume, calcium homeostasis, and possibly morphology (Szabo and Zoratti, 2014[77]). Electrophysiological techniques have identified different anion channels in the inner and outer mitochondrial membranes. VDAC (voltage-dependent, anion-selective channel) is an important outer mitochondrial membrane large conductance channel (∼4 nS in 1 M KCl) (De Pinto et al., 1987[29]) regulating metabolite flux across the membrane (Báthori et al., 1998[8]; Xu et al., 1999[84]). 

There is still very limited knowledge about the inner mitochondrial membrane chloride channels. For the first time, Sorgato et al. (1987[73]) by study of patched mitoplasts in brown fat mitochondrial revealed an anion-selective 107 pS channel (150 mM KCl) or centum picosiemens channel, mCS, that was not pH-dependent (pH 6.2-9.0). Subsequently, activation of an inner mitochondrial membrane anion channel (IMAC) was observed by mitochondrial swelling method. IMAC is permeable to single- and multi-charged anions, such as Cl^-^, ATP, citrate and superoxide (Beavis and Garlid, 1987[11]; Beavis, 1992[9]). The channel properties exhibit characteristic conductance (108 pS), bursting kinetics and pH-dependent. Several studies demonstrated that channel open probability (Po) decreased at low pH, whereas the channel activities switched to increased Po at alkaline pH (Schonfeld et al., 2004[68]; Borecky et al., 1997[17]). Electropharmacological properties of 108 pS channel, such as substate specificity, inhibitor specificity and pH dependent behavior, have been proposed that the channel might be identical to IMAC (Borecky et al., 1997[17]). The permeability transition pore (PTP) is another large conductance anion channel in mitochondrial inner membrane (De Marchi et al., 2006[27]; Szabó and Zoratti, 1991[78]). The channel characteristic exhibits a fast-gating behavior and maximal conductance 0.9-1.3 nS (150 mM KCl). PTP activity is strongly promoted by Ca^2+^ (Martinucci et al., 2000[55]). In this regard De Marchi et al. (2008[28]) characterized a voltage-dependent “maxi” mtCl channel in mitochondrial inner membrane of a colon tumor cell line. In the symmetrical 150 mM KCl, the channel conductance was around 400 pS which is half-conductance of the PTP. It was inhibited by several compounds including DIDS, SITS, and by ATP and Mg^2+^ at low pH. The mitochondrial megachannel (MCC) is the other candidate for mitochondrial inner membrane anion channel (Kinnally et al., 1989[46]). The channel exhibits nine conductance levels ranging from 40 pS to over 1,000 pS in symmetrical 150 mM KCl (Kinnally et al., 1996[47]; Zorov et al., 1992[91])

Chloride intracellular channel CLIC belongs to a newly described family of proteins that form anion channels in intracellular organelles and involvement in membrane trafficking (Jentsch et al., 2002[45]), apoptosis (Fernandez-Salas et al., 1999[31]) and cell differentiation (Suh and Yuspa, 2005[76]). CLIC4 seems localized in the mitochondria (Suh et al., 2004[75]) and may be a key element in the apoptotic response to oxidative stress (Xu et al., 2013[85]). 

In addition, brown adipose tissue mitochondrial uncoupling protein (UCP) reconstituted into giant liposomes displays stable chloride channel properties under patch-clamp conditions. Channel has a conductance of ~75 pS in symmetrical 100 mM KCl and closes at high positive potentials on the matrix side of UCP (Huang and Klingenberg, 1996[38]). Furthermore, electrophysiological study of the contact sides isolated from brain mitochondria by Moran et al. (1990[58]) showed that these particular fractions contain ion channels with conductances ranging from approximately 5 pS to 1 nS (in symmetrical 150 mM (KCl). 

In spite of a few reports to show that the chloride channels are present in brain mitochondrial inner membrane but its single channel behavior has not been well clarified. In this study, we show that rat brain mitochondrial inner membrane contains two voltage-gated 158 and 301 pS chloride channels that are sensitive to DIDS and NPPB but not to Ca^2+ ^ions. We also demonstrate that 301 pS but not 158 pS chloride channel is insensitive to ATP and acidic pH. Comparing the electro-pharmacological profile of 158 pS Cl channel to IMAC suggest that the channel is probably identical to IMAC and 301 pS Cl channel displays different properties than those classically described for mitochondrial anion channels.

## Materials and Method

HEPES, sodium bicarbonate, D-manitol, sucrose, digitonin, potassium chloride, Tris-HCl, BSA, nagarase, potassium chloride, EGTA, DIDS, ATP and NPPB were purchased from Sigma. n-Decane was obtained from Merck. Salts and all solvents were analytical grade. 

### Solutions

Solutions for mitochondrial isolation include the following: MSE solution (225 mM manitol, 75 mM sucrose, 1 mM EGTA, and 5 mM HEPES, 1 mg/ml BSA, pH 7.4); MSE-nagarse solution (0.05 % nagarse in MSE solution); MSE-digitonin solution (0.02 % digitonin in MSE solution).

### Mitochondria isolation

Mitochondria from two rat brains were isolated according to the protocol described by Rosenthal et al. (1987[64]). Subsequently, mitochondrial inner membranes were obtained as previously described (Da Cruz et al., 2003[26]). Briefly, brains of anesthetized rats homogenized in 20 ml ice-cold MSE-nagarase solution and was centrifuged at 2000 × g for 4 min. After supernatant centrifugation, the pellet was dissolved in 20 ml of MSE and digitonin. Then, the supernatant was centrifuged at 12000 × g for 11 min and pellet was dissolved in 300 μl of MSE solution. After suspending the mitochondria in H_2_O, the mixture was homogenized and the suspension was centrifuged twice at 12000 × g for 5 min. Mitoplasts were treated with Na_2_CO_3_ 0.1 M and suspension was centrifuged at 100000 × g for 30 minutes.

### L-α-Phosphatidylcholine extraction

L-α-Phosphatidylcholine (L-α-lecithin) was extracted from fresh egg yolk according to the protocol described by Singleton et al. (1965[72]) with small modifications. In brief, fresh egg yolks (500 g) were blended with acetone at 25 °C. After 1 hour and washing the solids three times with 200 ml portions of acetone 15 °C, solids were suspended in 600 ml 95 % ethanol, allowed to stand for 1 hour, and the mixture was then filtered. Thereafter, the ethanol extract was concentrated to dryness on an evaporator (LABOROTA 4000, Heidolph). The crude phospholipids were extracted with 100 ml petroleum ether and poured into 300 ml of acetone at 15 °C with rapid stirring. The precipitated phosphatides were washed with cold acetone. The petroleum ether-acetone step was repeated. After dissolving phosphatides in sufficient chloroform (5 % solution), solution was added to an alumina chromatographic column. The solvent system for eluting the phosphatide fractions was chloroform: methanol, 9:1 by volume. The very cloudy elutes was collected as L-α-Phosphatidylcholine. The progress of the fractionation was followed by thin layer chromatography.

### Electrophysiological studies

Bilayer lipid membranes (BLMs) were formed in a 200 μm diameter hole. The chambers contained 200/50 mM KCl (cis/trans) solutions using a suspension of L-α -lecithin in n-decane. Typical capacitance values ranged from 200 to 300 pF. BC-525D amplifier (Warner Instrument) was used to measure single channel currents. The *cis *chamber was voltage-clamped relative to the trans chamber, which was grounded. All recordings were filtered at 1 kHz and digitized at a sampling rate of 10 kHz. Single channel analysis was performed using the standard event detection algorithms in PClamp10. 

### Ethical considerations

All experiments were executed in accordance with the Guide for Care and Use of Laboratory Animals (National Institute of Health Publication No. 80-23, revised 1996) and were approved by the Research and Ethics Committee of Shahid Beheshti University of Medical Sciences (IR.SBMU.NRC.REC. 1388.30).

## Results

### Electrophysiological properties of two different anion channels

**Figure 1:**
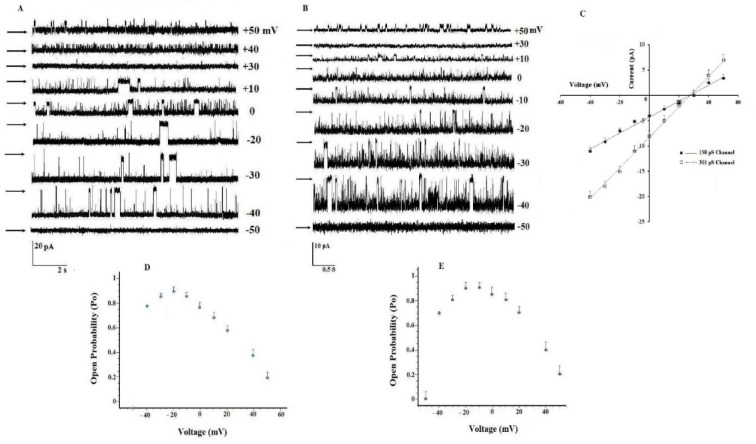
Single channel recordings, current/voltage curve and channel open probability as a function of voltages. (A and B) Single channel recordings in 200/50 mM KCl (cis/trans) at ±50 mV (the arrow indicates the closed state). (C) Current-volt age characteristics of single channel events in a 200/50 mM KCl (cis/trans) gradient solution, 301 pS channel (□) and 158 pS Channel (■). Error bars indicate the S.E. from 8 independent experiments. (D and E) Channel open probability (Po)-voltage curves.

After incorporation of the brain mitochondrial inner membrane vesicles, we usually observed two types of anion channels that had different single channel amplitudes and conductances (n = 55). Figure 1A and B(Fig. 1) presents two different examples of current traces obtained from brain mitochondrial inner membrane vesicles at different bilayer potentials, ranging from -40 mV to +50 mV. Current-voltage (I-V) plots were linear with no evidence of rectification at potentials between -40 mV and +50 mV (Figure 1C(Fig. 1)).These channels displayed a conductance value of 301 ± 8 pS (n = 8) and 158 ± 7 pS (n = 8) and negative reversal potentials close to +30 mV, which attest their anionic selectivity under our standard recording conditions consisting of 200 mM KCl cis/50 mM KCl trans. The open probabilities (Po) of 301 pS and 158 pS channels were also found to be voltage-dependent (Figures 1D and E(Fig. 1), respectively). Open probability of Cl channel as a function of voltages presented a plateau at ± 20 mV. Voltage above ± 50 mV almost inhibited all observed anion channels, substantially. Maximum value for Po was 0.9 ± 0.03 (n = 8). 

### Effect of Cl channel blockers

**Figure 2:**
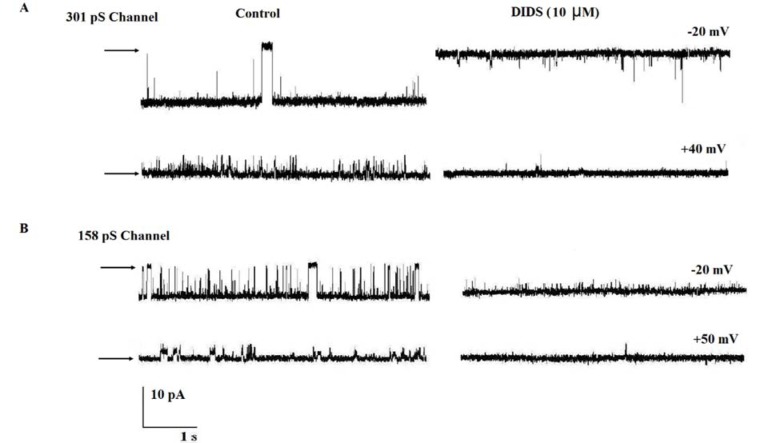
The effect of DIDS on channels gating behaviors at positive and negative voltages. (A and B) Single-channel recordings under control conditions (200/50 mM KCl; cis/trans) and immediately after cis addition of DIDS 10 µM (n = 4). Arrows indicate the closed levels.

**Figure 3:**
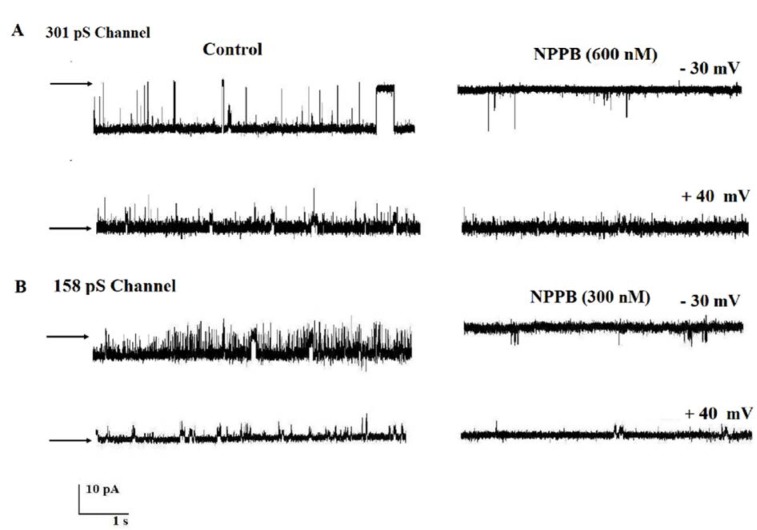
The effect of NPPB on channel activity at different voltages. (A and B) Representative recordings of channel currents under control conditions and after addition of 300 and 600 µM NPPB to cis face. Channels' activities are completely blocked at negative and positive potentials (n = 4). Arrows indicate the closed levels.

The channels' pharmacological properties were investigated by testing the effect of NPPB and DIDS. The application of 10 µM DIDS as nonspecific blocker to the cis compartment totally blocked the channels' activities at negative or positive voltages (Figures 2A and B(Fig. 2)). Cis face addition of 600 nM of NPPB inhibited the 301 pS channel activities at +40 mV and −30 mV (n = 4) (Figures 3A and B(Fig. 3)). In contrast, the results in Figure 3B(Fig. 3) demonstrate that the addition of NPPB 300 nM on the cis side of the reconstituted 158 pS anion channel activities inhibited the channel gating behaviors. 

These data indicate that the two Cl^−^ channels identified in this work display high sensitivities to these agents.

### Effect of ATP on 158 pS and 301 pS chloride channels activities

**Figure 4:**
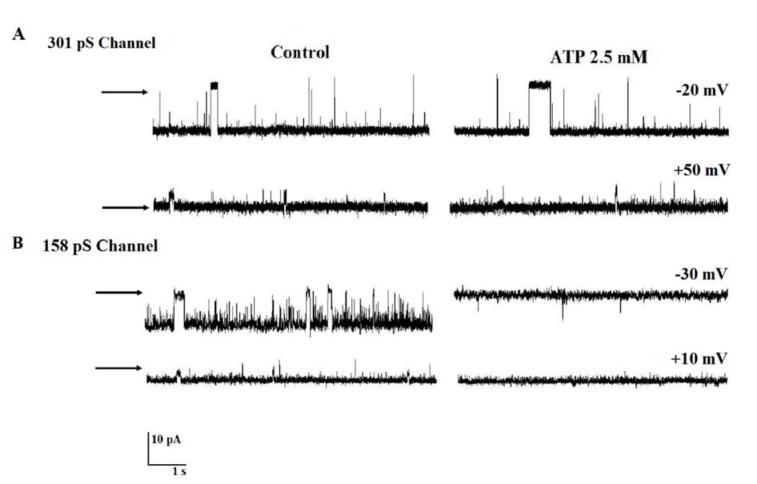
The effect of ATP on channels' gating behaviors. (A and B) Single channels' activities in control conditions and after adding 2.5 mM ATP to cis face (n = 4). The arrows indicate the closed levels.

To investigate whether nucleotide could regulate the two channels' activities, experiments were undertaken in which the action of ATP was measured on channels incorporated into lipid bilayers. As seen in Figure 4A(Fig. 4), the addition of 2.5 mM ATP to cis compartment did alter neither the channel conducting nor the channel gating behavior at +50 mV and -20 mV. In contrast, the addition of ATP 2.5 mM in cis chamber completely blocked 158 pS anion channel at negative and positive voltages. These results are representative of n = 4 on various membrane preparations.

### Effect of pH on channel activities

**Figure 5:**
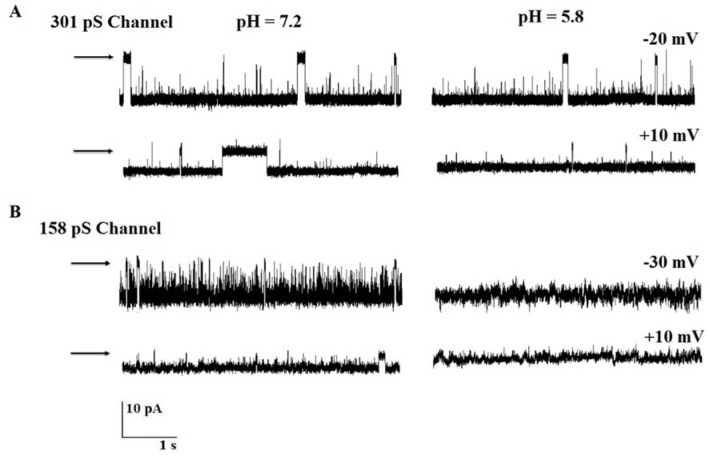
The effect of pH on anion channel activity. (A and B) Single channel recordings under control conditions (200/50 mM KCl; cis/trans, pH 7.2) and cis acidic pH (pH 5.8). Significant differences in the open probability value and amplitude of 158 pS anion channel are observed (n = 4). Arrows indicate the closed levels.

To further characterize these channels, the effect of acidic pH was examined on channels' activities. As seen in Figure 5A(Fig. 5), acidic pH (5.8) in the cis chamber did not alter either the channel conducting or gating behavior of 301 pS chloride channel at +10 mV and −20 mV (Figure 5A(Fig. 5), n = 4). In contrast, pH 5.8 resulted in an inhibition of the 158 pS anion channel activities at negative and positive voltages (Figure 5B(Fig. 5), n = 4).

### Effect of EGTA (Free calcium) on channel activity

**Figure 6:**
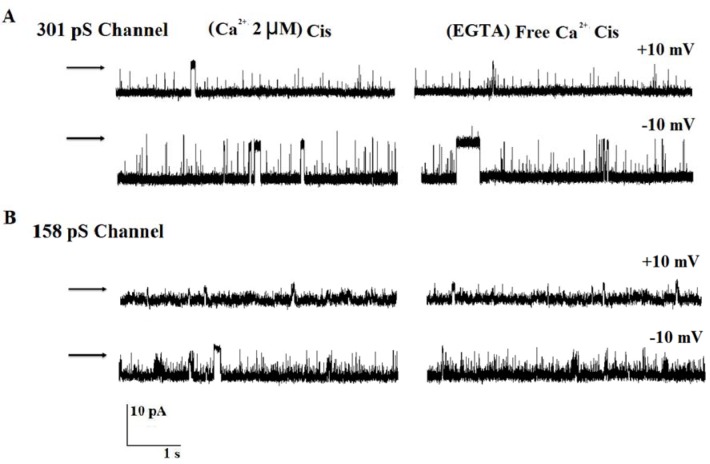
The effect of Ca^2+ ^ions on channel behavior. (A and B) Single-channel activities in control condition (10 µM calcium ions) and after addition of 1 mM EGTA (cis) in the same bilayer. Arrows indicate closed state of the channel.

To investigate the possible effect of Ca^2+^ on two chloride channels' gating behaviors, we recorded the channels activities in the absence of cis and trans Ca^2+^ ions. Figure 6(Fig. 6) shows single-channel recordings in gradient 200/50 mM KCl (cis/trans) solution at ±10 mV under 10 µM Ca^+2^ and calcium-free conditions in the same bilayer lipid membrane. Changes of two channels activity were not observed after addition of 1 mM EGTA to cis face. 

## Discussion

In this study we found that brain mitochondrial inner membrane has two different chloride channels.

Comparing the electro-pharmacological profiles determined in our study clearly argues against the candidacy of mitochondrial anion channels characterized by VDAC, mega chloride channels (MCC)/permeability pore transition (PTP) and UCP. Here, we describe that 158 pS ATP- and pH-sensitive anion channel is probably IMAC and a 301 pS ATP- and pH insensitive anion channel displays different properties than those classically described for mitochondrial anion channels. Electrophysiological techniques have been demonstrated various anion channels in inner and outer membranes. VDAC is an important voltage-dependent anion-selective channel in outer membrane regulating metabolite flux across the membrane (Báthori et al., 1998[8]; Schein et al., 1976[67]; Xu et al., 1999[84]). Purified VDAC has been characterized by reconstitution into planar lipid membrane (Benz, 1994[12]; Colombini, 1989[23]; Rostovtseva and Colombini, 1996[65], 1997[66]; Zizi et al., 1998[88]) as a large channel (∼4 nS in 1 M KCl) (De Pinto et al., 1987[29])*. *Under the influence of trans membrane voltages, reconstituted VDAC undergoes transitions in channel conductance and ion selectivity (Benz, 1994[12]; Colombini, 1989[23])*.* For example, at low voltages (< 10 mV), the channel is in its highest conducting state (open states) with selectivity to anions over cations, but at high voltages (> 40 mV), the channel converts to a lower conducting state (closed states) with higher selectivity to cations over anions (Benz, 1994[12]; Colombini, 1989[23]; Hodge and Colombini, 1997[37]). Although, both closed and open states exhibit ion selectivity, it is only weak (Schein et al., 1976[67]; Pavlov et al., 2005[60]). The properties of the channels described here are very different from those of the voltage-dependent anion channel (VDAC) of the outer mitochondrial membrane. VDAC represents a high conductance (>1 nS) and a poor Cl^−^/K^+^ selectivity (∼< 2) and lower conducting states observe at positive or negative voltages (Colombini, 1989[23]). The I/V relationship in Figure 1C(Fig. 1) demonstrates that the observed channels in this study are Cl selective channels, the Nernst reversal potential for K^+^ ions being equal to -34 mV. Furthermore, we did not observe high subconductance jumps of chloride current, as was reported for VDAC.

The mitochondrial permeability transition (Bernardi et al., 1999[14]; Gunter and Pfeiffer, 1990[33]; Zoratti and Szabo, 1995[90]), proposed to be a key early event in apoptosis (Bernardi et al., 1999[14]; Crompton, 1999[25]; Green and Reed, 1998[32]; Kroemer et al., 1998[49]; Marzo et al., 1998[56][57]; Scorrano et al., 1999[70]), is due to the opening of the 'permeability transition pore' (PTP) in the mitochondrial membrane system. Petronilli et al. (1989[61]) described in their study that application of negative or positive voltages on rat liver mitochondria inner membranes induced several different conductances, ranging up to 1.3 nS in symmetrical 150 mM KCl and at least those higher than 0.3 nS are substates of the highest conductance channel. Furthermore, matrix Ca (Haworth and Hunter, 1979[34]; Hunter and Haworth, 1979[39][40]; Hunter et al., 1976[41]) and depolarization (Scorrano et al., 1999[70]; Bernardi, 1992[15]) are two key features for PTP activity. Mitochondrial megachannel (MMC) has a high conductance of 0.9-1.5 nS range (150 mM KCl) with multiple substates including a half-conductance whose presence strongly suggests a dimeric structure (Avery et al., 1999[5]). Martinucci et al. (2000[55]) confirmed the identity PTP and MMC. Indeed, the possibility exists that different variants of the PTP might form, depending on experimental conditions. Therefore, there is question that whether our studied anion channels are different variants of PTP/MMC. A typical feature of the Po-voltage characteristics observed under control condition was their asymmetry in the voltage range between −50 mV and +50 mV. We showed a bell-shaped dependence of Po on voltage ranging from -40 mV to +40 with a narrow plateau at voltages ranging from −20 to +20 mV. Two Cl^-^ channels open probabilities inhibited at voltages above ±50 mV. This is in contrast to reported voltage-dependent activity of PTP or MMC (Haworth and Hunter, 1979[34]; Hunter and Haworth, 1979[39][40]; Hunter et al., 1976[41]). Bernardi (1992[15]) observed that open probability of rat liver mitochondrial membrane PTP increases at positive voltages, similar to what was reported on the mitochondrial PTP by Scorrano et al. (1997[69]). Furthermore, PTP represents a moderate permeability to anions (Cl^−^) over cations (K^+^), but it can switch to the opposite selectivity for limited periods (Campello et al., 2005[19]; De Marchi et al., 2006[27]). In contrast, the studied channels exhibited high discrimination between Cl^−^ and K^+^ (P_Cl−_/P_K+_ > 17). Another aspect of the present work concerns the effect of Mg^2+^ and Ca^2+^ on the brain inner mitochondrial anion channels, compared with PTP. Figure 6(Fig. 6) demonstrates that addition of 1 mM EGTA to both sides of the membrane did not affect channel activities. In addition, our experiments were done in the presence of ~1 mM Mg^2+^. It has been demonstrated that PTP activity is strongly promoted by Ca^2+ ^(Baines et al., 2003[6]; Cao et al., 2005[20]; Wang et al., 2005[82]) and inhibited by Mg^2+^. In all recording, we observed anion currents are independent and pharmacological profile differ significantly. We showed that the 158 pS anion channel is sensitive to ATP and acidic pH. Therefore, it does not seem that the 158 pS anion channel represents a long-lived substate or a monomer which dimerizes to form the 301 pS anion channel as were PTP. The three lines of evidence described above lead us to the conclusion that the studied channels are not different variants of PTP/MMC. 

An anion uniport pathway has been characterized in the inner mitochondrial membrane using flux measurements in mitochondria. The 108-pS channel was the first ion channel to be discovered in mitochondria by Sorgato et al. (1987[73]) and referred as the inner mitochondrial membrane channel (Zoratti and Szabo, 1994[89]) or mitochondrial centum picosiemens channel (mCS) (Ballarin and Sorgato, 1995[7]). The channel has been detected in patch-clamp experiments on mitoplasts from liver, heart (Sorgato et al., 1989[74]), brain (Moran et al., 1990[58]), and brown adipose tissue (BAT) and was characterized as only slightly anion selective (P_Cl−_/P_K+_ = 4.5), voltage sensitive and regulated by pH (Borecky et al., 1997[17]). The other anion channel from the inner mitochondrial membrane is IMAC (the mitochondrial inner membrane anion channel) (Beavis, 1992[9], Beavis and Garlid, 1987[11]), which is regulated by Mg^2+^ ions and the pH in the mitochondrial matrix. A decrease in matrix Mg^2+^ concentration or matrix alkalization enhances the permeability of the inner membrane to Cl^-^ (Beavis and Garlid, 1987[11]; Schonfeld et al., 2004[68]). The 107 pS mitochondrial inner membrane anion channel has been exhibited bursting behavior and an increased open probability at positive potentials.

It has been suggested that 108 pS channel reflects IMAC activity by applying patch-clamp to mitoplasts of BAT mitochondria (Borecky et al., 1997[17]) or reconstituted cardiac mitoplasts (Hayman et al., 1993[35]). The current-voltage relationships were ohmic at all voltages in our experiments and the mean conductances of our observed channels were estimated at 301 and 158 pS. According to our results, 158 pS Cl channel could be a candidate for the IMAC because of several properties. A conductance of 100-pS was demonstrated for the voltage-sensitive anion channel from mitochondria of both liver and cardiac tissues in 150 mM KCl in accordance with the present finding in asymmetrical condition (Sorgato et al., 1989[74]). The next line of evidence for the identity of these two types of channels is inhibitory effect of low pH. We demonstrated 158 pS Cl^-^ channel was inhibited by low pH, as were IMAC (Malekova et al., 2007[54]). In the current study, all experiments obtained in the present of ~1 mM free Mg^2+^ in cis and trans faces of the channel and no channel inhibition was observed.

This data is in contrast with Borecky et al. (1997[17]) studies who observed no channels with 108 pS characteristics by using patch clamp technique. On the other hand, when Sorgato et al. (1989[74]) reconstituted the 108 pS channel into a planar lipid membrane, they also recorded channel activities in the presence of 2 mM Mg^2+^. Taken together, it seems that the inhibition of mitochondrial anion channel by Mg^2+^ needs some other factors which have been lost when working with an isolated channel incorporated into a bilayer. Another aspect of the present work concerns the effect of EGTA; we observed the channel activities did not affect by EGTA. IMAC channels with similar feature were reported for channels identified in brown adipocytes by patch clamp in the mitoplast-attached mode with KCl solutions containing 2 mM EGTA and no added Ca^2+^ (Ballarin and Sorgato, 1995[7]). Furthermore, in accordance with our study, the inhibitory effects of DIDS (Beavis and Davatol-Hag, 1996[10]) and NPPB (Beavis, 1992[9]) on IMAC activities has been reported. Another piece of evidence for the identity of the 158 pS channel and IMAC comes from the finding of a similar pattern of ATP effect. Klitsch and Siemen (1991[48]) and others (Huang and Klingenberg, 1996[38]) showed that IMAC channel is inhibited by low concentrations of purine nucleotides; however, it is not identical with the uncoupling protein. In the current study, we showed the inhibitory effect of ATP on 158 pS channel activities. Thus, we conclude the possibility that channel activities recorded in our study is identical to IMAC. The only discrepancy was found in the voltage regulation of compared anion channels. Sorgato et al. (1987[73]) and Borecky et al. (1997[17]) described 108 pS channel was silent at membrane potentials below 0 mV and active at depolarizing potentials. In contrast, we showed a bell-shaped dependence of Po on voltage ranging from -50 mV to +50 mV. Our results are in line with Tomaskova et al. (2007[79]) studies who reported a 108 pS anion channel derived from mitochondrial membranes of the rat heart by planar lipid membrane technique. They showed a bell-shaped dependence of Po on voltage ranging from -60 mV to +100 mV with maximum Po at -20 to +60 mV. 

Uncoupling protein (UCP) family from brown adipose tissue is a membrane protein and a member of mitochondrial anion carrier family. Evidence from *in vivo* studies implicates the UCPs in the etiology of type 2 diabetes (Chan et al., 2001[21]; Lameloise et al., 2001[50]; Zhang et al., 2001[86]), in the mitigation of metabolic syndrome (Bernal-Mizrachi et al., 2002[13]; Clapham et al., 2000[22]), and in the mitigation of cellular damage due to reactive oxygen species (Arsenijevic et al., 2000[3]; Lee et al., 1999[51]; Li et al., 2001[52]; Negre-Salvayre et al., 1997[59]; Vidal-Puig et al., 2000[81]). UCP is the other candidate for a mitochondrial anion channel (Huang and Klingenberg, 1996[38]). Huang and Klingenberg (1996[38]) demonstrated the existence of a channel with a conductance of 150 or 75 pS in symmetrical 100 mM KCl. The channel conductance suggests that channel structure is either a dimer of two monomeric channels or a monomeric channel with 50 % subconductance state. Furthermore, it has been shown that UCP strongly discriminates against cations (P_Cl−/_P_K+_∼17) and is voltage sensitive, closed at high positive potentials on the matrix side of UCP. It should be mentioned that pH and divalent cations (Mg^2+ ^and Ca^2+^) do not affect channel activities (Huang and Klingenberg, 1996[38]).

There is question whether 301 pS anion channel could have something to do with the UCP. The similarities between the anion channel and the UCP we found that both channels have high anion selectivity, the same pH and divalent cation insensitivity. In addition, our results showed the Cl^-^ channel can be blocked by DIDS as UCP (Huang and Klingenberg, 1996[38]). The discrepancy was found in the ATP sensitivity and sub-conducting state of compared anion channels. UCP exhibited conductance of 75 pS for monomer and 150 pS for dimmer (Huang and Klingenberg, 1996[38]). We did not observe a single channel characterized by a sub-conducting state 50 % of the full unitary conductance. Figure 4(Fig. 4) showed ATP at 2.5 mM totally blocked 158 pS but not 301 pS anion channel (data not shown), our results exclude the possibility that 158 pS anion channel is a subconductance state corresponding to 50 % of the maximum unitary conductance (301 pS). Furthermore, nucleotide binding has served from the beginning as a tool for identifying the UCP (Heaton et al., 1978[36]; Jaburek and Garlid, 2003[42]; Lin and Klingenberg, 1980[53]). Taken together, we suggest that 301 pS anion channel does not belong to UCPs proteins.

The ADP/ATP carrier (AAC) is the most abundant membrane protein in mitochondria. Brustovetsky and Klingenberg (1996[18]) showed that this carrier can be converted reversibly into an anionic channel. 

Single channel recording of reconstituted mitochondrial ADP/ATP carrier (AAC) revealed a low cation selective (P_K+_/P_Cl_^-^ = 4.3 ± 0.6) channel with multiple subconductance levels from 300 to 600 pS. The channel gating behavior showed decreased open probability at voltage up to ±80-100 mV. The channel gating behavior is Ca^2+^- and pH-dependent and channel opening is inhibited at acidic pH and in the absence of Ca^2+^ (Brustovetsky and Klingenberg, 1996[18]). However, in our study, the 301 pS chloride channels were observed in the solutions containing EGTA, which blocks the AAC channel. Intracellular chloride channel family (CLIC) are localized in cellular compartments and expressed in multiple tissue types (Ashley, 2003[4]; Jentsch et al., 1999[44]). 

Very recently, it has been suggested that CLIC4 and CLIC5 localize to the outer and inner mitochondrial membrane (Ponnalagu et al., 2016[62]), respectively. CLIC5 plays a direct role in regulating mitochondrial reactive oxygen species (ROS) generation (Ponnalagu et al., 2016[62]). It has been suggested that CLIC4 forms a channel (conductance ~15 pS) that is poorly selective for chloride ions (with a mean Cl^-^/K^+ ^selectivity of 0.54 ± 0.09) and displayed several substates (Singh and Ashley, 2007[71]). The single channel properties of the chloride channels observed in our study were different from the reported CLIC4, which has a very low conductance (< 15 pS) and a poor Cl^−^/K^+^ selectivity (∼ < 2) (Singh and Ashley, 2007[71]). In addition, we did not observe subconductance jumps, as was reported for CLIC4. Taken together, 301 pS Cl channel displays different properties than those classically described for mitochondrial anion channels. Notably, despite numerous reports of mitochondrial chloride channel properties, their molecular origins and functional significances are still not fully understood. However, the complexity of the mitochondrial membranes did not permit a clear assignment of these permeabilities.

In conclusion, the results of our investigations show that brain mitochondrial inner membrane contains two types of chloride channels which exhibit 158 and 301 pS conductance, ATP- and pH-sensitive and insensitive properties, respectively. The 301 pS Cl^-^ channel described in this work shares no common features with other mitochondrial channels in terms of single-channel conductance and modulation by ATP. The blocking effect of ATP at physiological concentration on 158 pS Cl channel indicates that this channel is activated when the ATP concentration decreases. There is evidence to show that decreased concentration of ATP is produced where tissues are depleted of oxygen or metabolic nutrients (Dirnagl et al., 1999[30]; Jennings and Reimer 1991[43]; Trump and Berezesky, 1995[80]; Zhang et al., 2003[87]). In these processes, the ATP-chloride channel interactions observed in our study may play a role. A unique feature of these channels is their bell shape voltage dependent with a maximum Po at depolarizing potentials (±20 mV). It has been suggested that reactive oxygen species (ROS) induce the opening of anion channel (Aon et al., 2003[1], 2008[2]; Cortassa et al., 2004[24]) and mitochondrial membrane depolarization is related to ATP depletion (Wu et al., 1990[83]). A decrease in ATP production has been implied with increases in ROS and apoptosis (Ricci et al., 2003[63]). Therefore, we may speculate that decreased ATP concentration induces activation of mitochondrial chloride channels, thereby decreasing the membrane potential, activating the efflux of superoxide anion from the matrix and modulating apoptosis.

## Acknowledgement

This work was supported by a grant from the Neurophysiology Research Center of Shahid Beheshti University of Medical Sciences.

## Conflict of interest

The authors declare that they have no conflict of interest.
